# The gut-lung axis in the CFTR modulator era

**DOI:** 10.3389/fcimb.2023.1271117

**Published:** 2023-09-15

**Authors:** Florian Lussac-Sorton, Éléna Charpentier, Sébastien Imbert, Maxime Lefranc, Stéphanie Bui, Michael Fayon, Patrick Berger, Raphaël Enaud, Laurence Delhaes

**Affiliations:** ^1^ Univ. Bordeaux, Centre de Recherche Cardio-Thoracique de Bordeaux, INSERM U1045, Pessac, France; ^2^ INSERM, Centre de Recherche Cardio-thoracique de Bordeaux, Pessac, France; ^3^ CHU Bordeaux, Service de Parasitologie et Mycologie, Centre de Ressources et de Compétences de la Mucoviscidose (CRCM), Service de Pédiatrie, Service d’Exploration Fonctionnelle Respiratoire, CIC, Bordeaux, France

**Keywords:** gut-lung axis, microbiota, mycobiota, cystic fibrosis, CFTR modulators

## Abstract

The advent of CFTR modulators represents a turning point in the history of cystic fibrosis (CF) management, changing profoundly the disease’s clinical course by improving mucosal hydration. Assessing changes in airway and digestive tract microbiomes is of great interest to better understand the mechanisms and to predict disease evolution. Bacterial and fungal dysbiosis have been well documented in patients with CF; yet the impact of CFTR modulators on microbial communities has only been partially deciphered to date. In this review, we aim to summarize the current state of knowledge regarding the impact of CFTR modulators on both pulmonary and digestive microbiomes. Our analysis also covers the inter-organ connections between lung and gut communities, in order to highlight the gut-lung axis involvement in CF pathophysiology and its evolution in the era of novel modulators therapies.

## 1 Introduction

**Figure 1:**
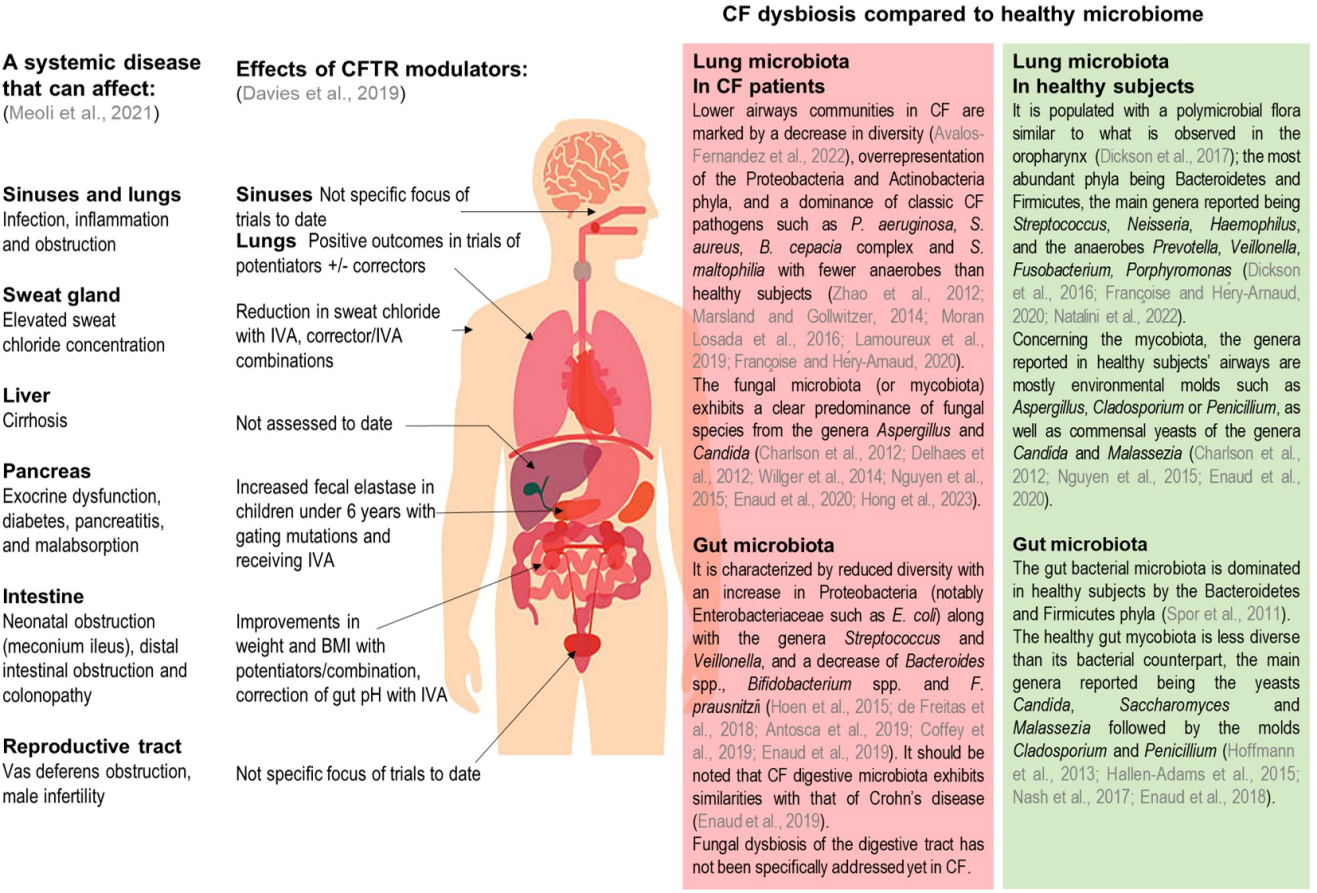
Summary of clinical manifestations, documented CFTR modulator effects, and of known lung and gut dysbiosis in pwCF. Adapted from Meoli et al., Pharmaceuticals 2021, and Davies et al., Kendig's Disorders of the Respiratory Tract in Children 2019. IVA, Ivacaftor; BMI, body mass index.

Cystic fibrosis (CF) is the most common severe life-limiting genetic disease in Caucasian populations. It affects more than 80,000 people worldwide, with a prevalence at birth of about 1 in 3,500 in Europe (Brown et al., 2017) but with a wide heterogeneity in its distribution. While it is diagnosed equally in men and women, its clinical expression appears to be more severe in women, which may be linked to estrogen involvement in the disease pathophysiology (Lam et al., 2021). CF is a monogenic disease with an autosomal recessive inheritance, caused by mutations in the gene coding the cystic fibrosis transmembrane conductance regulator (CFTR) protein, responsible for impaired chloride and bicarbonate secretions across epithelial cell apical membranes. More than 2,000 *cftr* gene mutations have been reported, which are classified into 6 groups according to the subsequent functional defect. These dysfunctions in the CFTR chloride channel cause an accumulation of viscous and dehydrated mucus in exocrine glands epithelia and airway lumen. While pulmonary complications remain the main cause of morbidity-mortality in CF, the disease also affects other organs in particular the gastro-intestinal tract (Karb and Cummings, 2021). The mucus accumulation may lead to an obstruction of the intestinal lumen as well as pancreatic and bile ducts. People with CF (pwCF) also exhibit an increased risk of gastro-intestinal cancers compared to general population (Yamada et al., 2018). Hence, CF represents a multi-organ pathology responsible for a wide variety of symptoms across patients’ lives (**Figure 1**), with respiratory and digestive manifestations playing key roles in disease progression.

In both the airways and gastro-intestinal tracts altered mucus composition leads to an imbalance in microbial communities. This dysbiosis also contributes to the establishment of chronic inflammation associated with functional lung and digestive decline (Enaud et al., 2019). Metagenomic approaches based on next generation sequencing have clearly facilitated the investigation of the CF microbiome (Françoise and Héry-Arnaud, 2020) which is of great importance to the understanding of the underlying mechanisms and to predict disease evolution. While assessment of both bacterial and fungal communities is well documented using metabarcoding approaches, the viral component of the microbiome requires shotgun metagenomics methods and remains largely unknown (Billard et al., 2017).

Pulmonary bacterial and to a lesser degree fungal microbiotas have been well documented in pwCF and healthy subjects (**Figure 1**) (Charlson et al., 2012; Delhaes et al., 2012; Zhao et al., 2012; Marsland and Gollwitzer, 2014; Willger et al., 2014; Nguyen et al., 2015; Dickson et al., 2016; Moran Losada et al., 2016; Dickson et al., 2017; Lamoureux et al., 2019; Enaud et al., 2020; Françoise and Héry-Arnaud, 2020; Avalos-Fernandez et al., 2022; Natalini et al., 2022; Hong et al., 2023). In the gut, microbial communities have also been well characterized (**Figure 1**) (Spor et al., 2011; Hoffmann et al., 2013; Hallen-Adams et al., 2015; Hoen et al., 2015; Nash et al., 2017; de Freitas et al., 2018; Enaud et al., 2018; Antosca et al., 2019; Coffey et al., 2019; Enaud et al., 2019); however it should be noted that fungal microbiota (also called mycobiota) has not been specifically addressed yet in pwCF digestive tract. Distant microbial florae display inter-organ connections involving a bidirectional crosstalk. As such, the gut-lung axis is an emerging concept describing how pulmonary and intestinal communities influence each other and are linked to clinical outcomes. In CF, this gut-lung axis is supported by studies results showing how gut bacterial microbiome is correlated with its pulmonary counterpart or the occurrence of respiratory complications (Enaud et al., 2020).

Before the 2010s, CF therapies were solely able to alleviate the disease symptoms. Over the last decade the development of CFTR modulators which directly address the underlying defects in the impaired protein has represented a turning point in the history of CF management (Davies et al., 2019). This has dramatically changed the disease’s clinical course (**Figure 1**) leading to meaningful improvements in the daily lives of a large CF population and enabling to reach an average life expectancy of nearly 50 years (Karb and Cummings, 2021). In this review we will focus on CFTR modulators, their impact on pulmonary and intestinal microbiotas and discuss how these novel therapies may modify profoundly the gut-lung axis in CF and which long-term clinical benefit CF patients may expect.

## 2 CFTR modulators

Current advances in CFTR modulators have been reviewed recently (Meoli et al., 2021). Here, we will focus on CFTR modulators for which microbiota data have been published. Briefly, Ivacaftor (IVA) (Kalydeco^®^, Vertex pharmaceuticals, Boston, Massachusetts) was the first modulator to obtain approval by the FDA (2012); it belongs to the “potentiator” class, which increases CFTR gating at the apical surface of epithelial cells allowing for extended channel opening time. IVA was first approved in adult patients with at least one G551D gating mutation, representing only 2% of CF patients (McKone et al., 2003). Its use has been secondarily extended to other rare CFTR mutations with gating defects and nowadays from the age of 4 months upwards. Its clinical efficacy was demonstrated in children and adult cohorts, with an improvement in lung function (*i.e*., increase of percent predicted forced expiratory volume in 1 second (ppFEV_1_)), a decrease in the rate of pulmonary exacerbations, an improvement in nutritional status (*i.e*., gain of weight and increase in body mass index (BMI)), an improvement in pancreatic function, and a strong reduction of the sweat chloride concentration (Ramsey et al., 2011; Davies et al., 2013). IVA displayed an acceptable safety profile (the most common adverse effects being headaches, oropharyngeal pain, upper respiratory tract infections and nasal congestion) (Ramsey et al., 2011; Davies et al., 2013). A risk of increased blood liver enzymes has been reported, justifying regular monitoring in all treated patients. No positive effects were observed when tested in patients with the more frequent F508del mutation (Flume et al., 2012), suggesting that a combination with a corrector is required to rectify the activity defect.

Several CFTR correctors have been developed to treat the protein misfolding and improve its trafficking to the apical surface, lumacaftor (LUM) being the first molecule of this class. Though LUM did not show any significant clinical effects as monotherapy (Clancy et al., 2012), it proved beneficial when associated with the potentiator IVA, and as such, has been FDA-approved as a combination since 2015 for patients aged 2 years and older, with homozygous F508del mutations (about 40% of pwCF (Wainwright et al., 2015)). The combination of lumacaftor-ivacaftor (LUM/IVA) (Orkambi^®^,Vertex pharmaceuticals) was evaluated in two randomized controlled trials, which showed modest but significant benefits on lung function at 24 weeks and on nutritional status (increase in BMI) (Wainwright et al., 2015). These benefits continued to be observed in the long term at week 96 (Konstan et al., 2017). LUM/IVA combination displayed an acceptable safety profile in the pivotal trials. However, clinical responses may also vary significantly amongst patients with the same genotype, and acute respiratory events (chest tightness, dyspnea and drop of ppFEV_1_) were reported later on in real life studies, responsible for treatment discontinuation in approximately 20% of patients (Hubert et al., 2017; Jennings et al., 2017; Labaste et al., 2017; Burgel et al., 2020).

The second dual-combination of a CFTR corrector and potentiator was tezacaftor-ivacaftor (TEZ/IVA) (Symdeko^®^ in the USA and Symkevi^®^ in Europe, Vertex pharmaceuticals), FDA-approved in 2018 for patients aged 6 years and older, with homozygous F508del mutations or heterozygous in association with a residual-function mutation. TEZ/IVA combination showed significant clinical efficacy similar to that of LUM/IVA, but a better side-effect profile with less occurrence of acute respiratory events (Rowe et al., 2017; Taylor-Cousar et al., 2017; Lommatzsch and Taylor-Cousar, 2019). Thereafter, a triple-combination approach was developed by adding elexacaftor (ELX), a new CFTR corrector, to the former TEZ/IVA regimen. ELX and TEZ bind to different sites of the CFTR protein, displaying an additive effect to improve the protein processing. The triple-combination elexacaftor-tezacaftor-ivacaftor (ELX/TEZ/IVA) (Trikafta^®^ in USA and Kaftrio^®^ in Europe, Vertex pharmaceuticals) was approved in 2019 for patients aged 6 years and older having at least one F508del mutation (regardless of the other mutation on the second allele), representing nearly 90% of pwCF (Cystic Fibrosis Foundation, 2022; Vaincre la Mucoviscidose, 2022). ELX/TEZ/IVA has been assessed in several randomized clinical trials (Heijerman et al., 2019; Middleton et al., 2019; Zemanick et al., 2021) which demonstrated an unprecedented clinical improvement with an acceptable safety profile leading to a significant decrease in the number of lung transplantations (Burgel et al., 2021).

## 3 Pulmonary microbiota

CFTR modulators therapies lead to improved mucus hydration by correcting chloride channel activity, which improves mucociliary clearance and, in turn, induces changes in airway microbial communities. The impact of modulators on the pulmonary microbiome has been more extensively studied during IVA monotherapy (as the first developed molecule) in patients bearing at least one G551D mutation. Results of these studies are summarized in **Table 1**. Briefly, a first study assessing IVA effects reported no significant changes in bacterial alpha-diversity, but a trend towards decreased abundance of pathogens as well as a significant increase in the anaerobe *Prevotella* (Rowe et al., 2014). A subsequent study performed on 3 patients did not find any significant differences in total bacterial load and global community composition, but described a notable decrease in *Streptococcus mitis* abundance and increase in the anaerobe *Porphyromonas* (Bernarde et al., 2015). A third study reported a significant increase of bacterial diversity indices during the first year of treatment in patients chronically infected with *P. aeruginosa*, as well as a decrease in the relative abundance of *P. aeruginosa* with reciprocal increase of oropharyngeal bacteria such as *Streptococcus* and *Prevotella* (Hisert et al., 2017). Nonetheless, these changes were not sustained with a notable rebound observed during the second year of IVA monotherapy (Burgel et al., 2021). A more recent study reported increased richness and diversity of the pulmonary bacterial microbiome, correlated with lower levels of circulating inflammatory markers (Einarsson et al., 2021). By contrast, other studies did not find any significant changes in pulmonary bacterial microbiome related to IVA in patients with G551D or S1251N mutations (Peleg et al., 2018; Harris et al., 2020; Kristensen et al., 2021), in spite of limitations due to small sample sizes, short follow-up periods and differences in antibiotic exposures. Overall, these results suggest a trend towards increased bacterial diversity in the airways on IVA monotherapy, accompanied with a decrease but no eradication of *P. aeruginosa* and reciprocal increase in commensal anaerobes.

**Table 1:** Impact of CFTR modulators on pulmonary and digestive microbiomes.

CFTR modulators assessed	Authors	Studied population	Follow-up period	Effects on bacterial microbiota	Effects on fungal microbiota
PULMONARY MICROBIOME					
IVA	Rowe et al., 2014	133 patients (with microbiome analysis limited to 14 patients), age 6 and older, at least one G551D mutation	6 months	Trend toward a decrease in relative abundance of CF pathogens. Significant increase in *Prevotella* relative abundance. Reduction of *P. aeruginosa* isolation from respiratory cultures in the whole 133 patient’s cohort.	NA
	Bernarde et al., 2015	3 patients, age range 10-16, at least one G551D mutation	mean 10 months	No significant changes in global community composition. Decrease in *Streptococcus mitis* relative abundance and increase of *Porphyromonas*.	NA
	Hisert et al., 2017	12 patients (microbiome analysis in 8 patients), age range 22-57, at least one G551D mutation	≥ 2 years	Increase of alpha-diversity during the first year, with decrease in *P. aeruginosa* relative abundance and reciprocal increase of commensal bacteria such as *Streptococcus* and *Prevotella*. Reduction in *P. aeruginosa* loads (assessed by culture and qPCR) in the first year. Rebound during the second year.	NA
	Peleg et al., 2018	20 patients, age range 18-65, at least one G551D mutation	4 weeks	No significant changes in microbial composition. In subjects with stable antibiotic exposure, reduction in total bacterial load.	NA
	Harris et al., 2020	31 patients, age 10 and older, at least one G551D mutation	6 months	No significant changes in diversity and bacterial loads (total and *P. aeruginosa*).	NA
	Kristensen et al., 2021	16 patients, mean age 22.5, at least one S1251N mutation	2-12 months	Trend toward an increase in alpha-diversity. No significant changes in overall microbial composition.	NA
	Einarsson et al., 2021	14 patients, age range 13-39, at least one G551D mutation	mean 1 year	Increase in alpha-diversity, correlated with lower levels of circulating inflammatory markers.	NA
LUM/IVA	Graeber et al., 2021	14 patients, age range 12-41, F508del homozygous	8-16 weeks	Decrease in total bacterial load and increase in alpha-diversity (with reduced IL-1β concentration in sputa).	NA
	Neerincx et al., 2021	16 patients, median age 25, F508del homozygous	1 year	Temporary and moderate decrease in *P. aeruginosa* relative abundance (statistically unsignificant, not sustained after 1 year).	NA
	Hong et al., 2023	66 CF patients (18 treated with LUM/IVA and 6 with IVA), age 18 and older	no longitudinal follow-up	NA	Higher alpha-diversity compared to untreated patients
	Enaud et al., 2023	41 patients, age 12 and older, F508del homozygous	6 months	No significant changes in diversity and bacterial loads (total and *P. aeruginosa*). In a subgroup of patients uncolonized with *P. aeruginosa* at baseline, increase in alpha-diversity.	No significant changes in diversity and total fungal load.
ELX/TEZ/IVA	Pallenberg et al., 2022	31 patients, age range 12-44, at least one F508del mutation	3-12 months	Increase in alpha-diversity (evenness) and beta-diversity. Decrease in total bacterial load and relative abundances of *P. aeruginosa* and *S. aureus*.	NA
	Sosinski et al., 2022	24 patients, mean age 32, at least one F508del mutation	mean 6 months	Increase in alpha- and beta-diversity. Decrease of the abundance log-ratio of CF pathogens/anaerobes.	NA
	Schaupp et al., 2023	65 patients, age 12 and older, at least one F508del mutation	1 year	Increase in alpha-diversity, correlated with reduced inflammatory markers in sputa. Decrease in *P. aeruginosa* relative abundance at 3 months.	NA
DIGESTIVE MICROBIOME					
IVA	Ooi et al., 2018	16 patients, age range 5-50, at least one G551D or G178R mutation	median 6 months	Increase in *Akkermansia* relative abundance. Moderate decrease in *Enterobacteriaceae* abundance (statistically unsignificant), correlated with reduced fecal calprotectin.	NA
	Kristensen et al., 2021	16 patients, mean age 22.5, at least one S1251N mutation	2-12 months	Significant increase in alpha- and beta-diversity. No significant changes in specific genera abundances.	NA
	Pope et al., 2021	12 patients (with pancreatic sufficiency), age range 21-64, at least one R117H mutation	4.4 months	No significant changes in diversity and microbial composition.	NA
	Ronan et al., 2022	14 patients, age range 18-39, at least one G551D mutation	median 1 year	No significant changes in diversity and microbial composition.	NA
LUM/IVA	Pope et al., 2021	8 patients (with pancreatic insufficiency), age range 8-24, F508del homozygous	median 7 months	No significant changes in alpha-diversity. Trend toward microbial composition closer to patients with pancreatic sufficiency.	NA

IVA, Ivacaftor; LUM/IVA, Lumacaftor + Ivacaftor; ELX/TEZ/IVA, Elexacaftor + Tezacaftor + Ivacaftor; NA, not assessed.

Regarding the impact of the LUM/IVA dual-combination on the airway microbiome, a first study reported similarly a moderate decrease in *P. aeruginosa* relative abundance that did not reach statistical significance and was not sustained after 12 months of treatment (Neerincx et al., 2021). A second study described a significant decrease in total bacterial load and an increase in bacterial alpha-diversity calculated with the Shannon index, along with a reduced concentration of the proinflammatory cytokine IL-1β in pwCF sputa (Graeber et al., 2021). Regarding the mycobiome, one recent cross-sectional study showed that adult patients treated with modulators (the majority of whom were receiving LUM/IVA) had significantly higher fungal alpha-diversity compared to untreated patients (Hong et al., 2023). A last study did not find any significant differences in both airway bacterial and fungal microbiota and pulmonary inflammation assessed by calprotectin measurement (Enaud et al., 2023). However, when focusing on a subgroup of patients uncolonized with *P. aeruginosa* at LUM/IVA initiation, calprotectin levels were lower and a significant increase in bacterial alpha-diversity was observed after 6 months of therapy, suggesting that microbiome changes on LUM/IVA are dependent on *P. aeruginosa* colonization status.

Only three studies have addressed the effect of the most recent and promising ELX/TEZ/IVA triple-combination on pulmonary microbiome in pwCF wearing at least one F508del mutation. One study performed on 24 patients (Sosinski et al., 2022) demonstrated a significant increase in bacterial alpha- and beta-diversity in the sputa after treatment, as well as a trend towards a reduced bacterial load. Although the relative abundances of specific bacterial taxa were unchanged, the authors described a significant decrease in the CF pathogens to anaerobes log-ratio. These findings were partially confirmed by a subsequent study reporting a significant increase in the evenness of distribution of bacterial taxa, along with a decrease in total bacterial load and relative abundances of major CF pathogens *P. aeruginosa* and *Staphylococcus aureus* (Pallenberg et al., 2022). Similarly, the authors of a recent study described an increase in bacterial alpha-diversity after 1, 3 and 12 months on ELX/TEZ/IVA (Schaupp et al., 2023) while the decrease in *P. aeruginosa* relative abundance was only significant at 3 months. In addition, the authors also reported a significant reduction in inflammatory markers (IL-1β, IL-8 and neutrophil elastase) in the sputa. All these findings appear to be consistent with those reported with previous generations of CFTR modulators.

## 4 Digestive microbiota

To date the impact of CFTR modulators on the intestinal microbiome has been less extensively studied compared to its pulmonary counterpart. Several studies suggest that IVA monotherapy may have an impact on the intestinal microbiome in pwCF with gating defects; such results are summarized in **Table 1**. A first study showed that IVA provokes a significant increase in the relative abundance of the bacterial genus *Akkermansia* (Ooi et al., 2018) which is known for its anti-inflammatory effects and as a biomarker of healthy gut mucosa (Derrien et al., 2017; Rodrigues et al., 2022). This finding was associated with a significant decrease in intestinal inflammation assessed by fecal calprotectin measurement, while bacterial alpha- and beta-diversities were unchanged. Conversely, another study reported a significant increase in both alpha- and beta-diversity indices after IVA treatment, whereas no significant changes were found in specific bacterial genera abundances (Kristensen et al., 2021). Two other studies did not show any significant effect related to IVA monotherapy on the gut microbiome (Pope et al., 2021; Ronan et al., 2022). Finally, trends toward a bacterial microbiota composition closer to subjects with normal exocrine pancreatic function were reported with LUM/IVA dual-combination (Pope et al., 2021). These limited findings need to be further validated by larger studies, especially with next-generation modulators like the ELX/TEZ/IVA triple-combination. Moreover, it must be noted that the intestinal mycobiota has not been assessed to date in patients treated with CFTR modulators. By extrapolation based on similarities regarding the bacterial component (Enaud et al., 2019) it may display similarities with patterns reported in inflammatory bowel diseases, which are characterized by a notable increase in the Basidiomycota/Ascomycota ratio during flares episodes and a decrease during remission (Sokol et al., 2017). In addition, although the intestinal mycobiome has been reported to influence airway outcomes (Zhang et al., 2017) no studies have currently examined this hypothesis or the role of the gut in the context of CF.

## 5 Discussion and perspectives: towards a gut-lung axis analysis in pwCF on CFTR modulators

In this review we have summarized the published data regarding the impact of CFTR modulators on pulmonary and digestive microbiomes respectively. However, these two microbial florae are not strictly independent as indicated by growing evidence describing inter-organ connections (Enaud et al., 2020; Françoise and Héry-Arnaud, 2020), and supporting the concept of a gut-lung axis. Due to the anatomical links the microbial communities of both tracts have direct interactions through gastroesophageal content inhalations and sputum swallowing (Enaud et al., 2020). Long-reaching interactions between the airways and intestine are also involved, mediated via the mesenteric lymphatic system through which microbial metabolites are exchanged (Bingula et al., 2017; Anand and Mande, 2018; Enaud et al., 2020). Among them, short-chain fatty acids synthesized by intestinal bacteria are of particular importance given their well-known immunomodulatory properties. Such fatty acids have been shown to mitigate the airway inflammatory response (Trompette et al., 2014; Halnes et al., 2017).

In pwCF, several studies suggest interactions between bacterial communities at the pulmonary and digestive levels. In infants with CF, a high degree of concordance was observed between bacterial genera evolution over time at both sites, with some genera colonizing the gut prior to their appearance in the respiratory tract (Madan et al., 2012). Furthermore, the gut microbiome is closely related to CF respiratory complications. Indeed, some gut microbiota alterations (such as a decrease of *Parabacteroides*) precede the onset of *P. aeruginosa* colonization, while its composition is associated with CF exacerbation in early life (Hoen et al., 2015). Regarding dietary exposures, breastfeeding was associated with microbial diversity of the respiratory tract as well as a prolonged time to first CF exacerbation (Madan et al., 2012; Hoen et al., 2015), the role of short-chain fatty acids being discussed but not demonstrated yet. Finally, the oral administration of probiotics (supposed to modulate the gut microbiota) showed a significant reduction in the number of pulmonary exacerbations, although the size of effect is unclear (Anderson et al., 2017).

The gut-lung axis involvement in pwCF receiving CFTR modulators has been far less extensively investigated. To date, only one study has assessed bacterial communities in both the airways and gut in 16 patients with the rare S1251N mutation treated with IVA monotherapy (Kristensen et al., 2021). The authors reported significant improvements in the gut microbiota diversity that were not observed in the respiratory samples (including sputum, nasopharynx and oropharynx samples). Nonetheless, these findings are limited by the small sample size (n=16, among which only 8 patients were followed during 12 months). In addition, the authors suggested a follow-up time in excess of 12 months to detect significant improvements in lung microbiota on CFTR modulators, since the development of respiratory tract microbiota appeared to be presaged by gut colonization (Madan et al., 2012).

Further studies are thus required to fully decipher the gut-lung axis evolution on modulator therapies, involving a joint analysis of pulmonary and fecal samples. Moreover, microbiome analyses have so far been almost exclusively limited to the bacterial kingdom, the fungal kingdom being neglected in the assessment of pwCF microbiotas on modulators, especially in the digestive tract analysis. In addition, the use of shotgun metagenomics approaches will allow to investigate the viral component of the microbiome, providing an exhaustive inter-kingdom assessment of CF microbial communities. The entire microbiome data should also be correlated with clinical parameters and surrogate inflammatory biomarkers for a comprehensive evaluation of the CF disease landscape in a personalized medicine approach that next generation of CFTR modulators will enable (Yi et al., 2021; Enaud et al., 2023).

Regarding the populations studied, it should be noted that the patients included in the quoted studies were over 5 years of age, including a majority of adults with already established chronic colonization and infection (**Table 1**). The modulators’ impact should also be assessed in future studies in large pediatric populations including children under 5 years of age, since CFTR modulator treatment will be initiated earlier, before the patients are chronically colonized and the gut-lung axis mucosa irreversibly damaged (Enaud et al., 2023). Moreover, other features such as pwCF gender should be considered, since differential outcomes between men and women treated with IVA have been described (Secunda et al., 2020). Finally, early published studies being focused on the assessment of patients treated with IVA monotherapy, future studies are needed to investigate the gut-lung axis in patients receiving combinations of modulators such as LUM/IVA, TEZ/IVA and ELX/TEZ/IVA.

In conclusion, the advent of CFTR modulators marks a turning point in the history of CF management and outcome, with unprecedented clinical improvements and favorable safety profiles, even if data about long-term adverse effects are not yet available. The impact of such molecules on the resident microbial communities of the respiratory and digestive tracts has only been partially deciphered, mostly with first-generation therapies such as IVA. While the results of available studies are not totally consistent, they suggest that CFTR modulators may induce an increase in bacterial diversity, associated with a decrease in conventional culture-based CF pathogens in the airways and an increase in anti-inflammatory bacteria in the gut. The impact of next-generation modulators on the gut-lung axis will have to be more extensively investigated in future studies, with additional data regarding the mycobiota and virobiota, especially in younger pwCF who will benefit even more from these innovative therapies.

## Author contributions

FL-S: Conceptualization, Writing – original draft, Writing – review & editing. ÉC: Conceptualization, Writing – review & editing. SI: Conceptualization, Writing – review & editing. ML: Writing – review & editing. SB: Writing – review & editing. MF: Writing – review & editing. PB: Writing – review & editing. RE: Conceptualization, Writing – review & editing. LD: Conceptualization, Writing – original draft, Writing – review & editing.
